# The Role of Early Molecular Response in the Management of Chronic Phase CML

**DOI:** 10.1007/s11899-017-0375-0

**Published:** 2017-04-12

**Authors:** Patrick Harrington, Aytug Kizilors, Hugues de Lavallade

**Affiliations:** 1 Department of Haematological Medicine, King’s College Hospital NHS Foundation Trust, London, UK; 2 Haematology Department, King’s College London, London, UK; 3 Laboratory for Molecular Haemato-Oncology, King’s College Hospital NHS Foundation Trust/King’s College London, London, UK

**Keywords:** Tyrosine kinase inhibitors, CML, Chronic myeloid leukemias, Early molecular response

## Abstract

**Purpose of Review:**

Although tyrosine kinase inhibitors (TKIs) spectacularly improve the disease burden and the overall survival of chronic myeloid leukemia patients, early identification of a subset of poor TKI responders has been recognized as a critical goal to prevent disease progression in these patients. We herein review the past and recent evidence on the impact of early response.

**Recent Findings:**

In the recent years, the achievement of an early molecular response (EMR, defined as 3-month *BCR-ABL1* transcript <10% IS) has emerged as a useful tool to identify poor-risk patients. Although several groups have reported the importance of such milestone, clinical intervention based on it remains controversial partly due to its low specificity to predict progression, which may be partially improved by using the rate of decline in *BCR-ABL1* transcript level (halving time or velocity of ratio reduction).

**Summary:**

Standardization of halving time or velocity of ratio reduction will likely help establishing more stringent recommendation and modify current clinical practices.

## Introduction

Chronic myeloid leukemia (CML) is a clonal myeloproliferative neoplasm originating from a single pluripotent hematopoietic stem cell, in which cells of the myeloid lineage undergo inappropriate clonal expansion caused by a molecular lesion. CML is characterized by the occurrence of the Philadelphia chromosome, which results from the fusion of the breakpoint cluster region (BCR) gene on chromosome 22 and the Abelson murine leukemia viral oncogene homolog 1 (ABL1) gene on chromosome 9. This generates the *BCR-ABL1* oncogene that encodes for a chimeric but active oncoprotein, the BCR-ABL tyrosine kinase; its deregulated activity is necessary and sufficient for malignant transformation [1]. The disease typically progresses through three distinct phases—chronic phase, accelerated phase, and blast crisis—during which the leukemic clone progressively loses its ability to differentiate [1, 2].

Since their introduction in 2001, tyrosine kinase inhibitors (TKIs) targeting BCR-ABL have become the standard therapy for CML. While allogeneic hematopoietic stem cell transplant (Allo-HSCT) is a recognized curative treatment for CML [3], TKIs prevent progression to advanced phase in most patients and spectacularly improve the disease burden and the overall survival of CML patients [4–8].

At present, five TKIs are approved for the treatment of CML: imatinib (a first-generation TKI), nilotinib, dasatinib, bosutinib (all three second-generation TKIs), and ponatinib (a third-generation TKI). The first three compounds are approved for the treatment of newly diagnosed patients who are treatment-naïve, while bosutinib and ponatinib are indicated in patients with intolerant or resistant CML.

Although cytogenetic responses had originally been the gold standard to assess treatment response in CML patients, *BCR-ABL1* transcript level by quantitative PCR assays (RQ-PCR) has become the reference in the last two decades and international collaboration has allowed harmonization of protocol and reporting of results [9]. Expert panels recommended that residual disease should be expressed on an International Scale (IS) based on standard values [10], and more recently, criteria for deep molecular responses have been established, introducing different levels of molecular response including MR4 (equivalent to 4 log reduction), MR4.5 (4.5 log reduction), and MR5 (5 log reduction) [11••].

Correspondence between complete cytogenetic response (CCyR) and BCR-ABL <1% IS (2-log response/MR2) and major cytogenetic response (MCyR) and *BCR-ABL1* transcript level <10% IS has since then been recognized, although concordance is not fully established [12].

## First Reports on the Impact of Early Responses on Treatment Outcome

Recognition of the importance of an early response to treatment in CML predates the TKI era, with Mahon et al. publishing data in 1998 demonstrating the importance of achieving a complete hematologic response after 3 months of treatment with interferon [13]. The significance of early molecular response to TKI therapy was first noted in 2002 by Merx et al., who found that an early response to imatinib, with *BCR-ABL1* transcripts levels reducing to 20% of the baseline value within 2 months of initiation of treatment, was predictive of major cytogenetic response [14]. Shortly after Wang et al. reported on the importance of achieving a 50% reduction in transcript level after 4 weeks and to less than 10% after 3 months, showing higher probability of achieving MCyR at 6 months and superior progression free survival after a follow up of 16.5 months [15].

In a cohort of 204 newly diagnosed chronic phase (CP)-CML patients treated with imatinib, we reported the impact of early cytogenetic response at 3 and 6 months [16]. A 5-year cumulative incidence of complete cytogenetic response (CCyR) of 96.4% was identified for those with a major cytogenetic response at 3 months, compared with 90.4% for those with a minor cytogenetic response and 30.8% for patients with no cytogenetic response. Similarly, a major cytogenetic response at 6 months was associated with a 98% 5-year cumulative incidence of CCyR, compared with 91.8% for those with a minor cytogenetic response and 25.4% for patients with no cytogenetic response.

## Early Molecular Response—Seminal Studies

As the use of real time quantitative polymerase chain reaction (RQ-PCR) techniques for monitoring *BCR-ABL1* transcript level began to supersede traditional cytogenetic monitoring and became more a commonplace in regular clinical practice, it was increasingly used to further demonstrate the importance of an early response to TKI therapy.

The impact of molecular response at 6 and 12 months in patients treated with first-line imatinib was first reported by Hughes et al. in the cohort of patients treated within IRIS (International Randomized Study of Interferon and STI571) [17]. Among 476 patients with PCR assessment, patients with *BCR-ABL1* transcripts >10% at 6 months and >1% at 12 months had inferior event-free survival (EFS) (7-year probability of EFS at 56.3% for patients with *BCR-ABL1* transcripts >10% at 6 months) and higher rates of progression to advanced phases.

The first publication on the impact of early molecular response (EMR, defined as 3-month *BCR-ABL1* transcript <10% after conversion to the international scale) were reported by Marin et al. who analyzed data from 282 consecutive newly diagnosed CP-CML patients treated with front-line imatinib. *BCR-ABL1* transcript level at 3, 6, and 12 months strongly predicted overall survival, progression free survival, and event free survival, as well as correlating with the likelihood of achieving CCyR, major molecular response (MMR), and complete molecular response (CMR) [18••]. Specifically, they identified that a transcript level of >9.84% after conversion to the IS at 3 months was associated with an 8-year probability of overall survival of 56.9% compared to 93.3% in those patients achieving a transcript level below this cutoff (*P* < 0.001). Sixty-eight patients (24%) failed to achieve this target and this was found to be the only independent predictor for overall survival in a multivariate analysis. The use of transcript cutoffs at 6 and 12 months did not predict any new cytogenetic failures or deaths and was considered to be both less sensitive and specific.

Similarly, Hanfstein et al. demonstrated the importance of an early molecular response in a cohort of 1303 patients on first-line imatinib treatment as part of the German CML IV study [19••]. They looked at three different risk groups defined by *BCR-ABL1* transcript level at 3 months. Those with a transcript level >10% IS represented 28% of the patient group, a similar figure to that seen in the study by Marin et al., and had a 5-year overall survival (OS) of 87%. Those with a transcript level of 1–10% IS accounted for 41% of patients and had a 5-year OS of 94% (*P* = 0.012), and the remaining patients with a transcript level of <1% IS had a 5-year OS of 97%. Despite the slightly better OS rates in the <1% IS group compared with the 1–10% IS group, there was no statistical difference identified between outcomes, and as such, the 10% cutoff was chosen as the most relevant landmark to denote a high-risk group.

## Early Molecular Response and Second-Generation TKIs

In view of the increased recognition of the efficacy of second-generation TKIs, Marin et al. investigated the relevance of EMR in patients treated with first-line dasatinib as part of the UK SPIRIT2 clinical trial [20•]. They reported that 8.6% of the 142 patients treated with first-line dasatinib would not achieve EMR as defined by *BCR-ABL1* transcripts of <10% IS at 3 months. The 2-year cumulative incidence of CCyR was only 58.8% for those who failed to achieve EMR compared to 96.6% of those who met the EMR target. Similarly, EMR predicted for 2-year cumulative incidence of MR3 (14.3 vs 79.8%, *P* < 0.001) and MR4.5 (0 vs 45.7%, *P* < 0.001).

The DASISION study directly compared first-line dasatinib and imatinib treatment, with 519 patients randomized on a 1:1 basis between the two treatments [12]. Dasatinib was associated with a significantly higher 3-month EMR rate with 84% of patients achieving the <10% IS target, compared with 64% in the imatinib group (*P* < 0.0001). This translated into improved progression-free survival (PFS) for patients reaching EMR in the imatinib arm (96 vs 75%) as well as in patients treated with dasatinib (93 vs 68%).

However, the overall survival and progression-free survival were not significantly higher in the dasatinib arm. It was suggested that this may have been due to the fact that it was more difficult to rescue dasatinib refractory patients.

Hughes et al. subsequently published data showing superior rates of EMR among those treated up front with the other second-generation TKI nilotinib, compared with imatinib [21]. Eight hundred forty-six patients were included with 33% in the imatinib arm failing to achieve a transcript level of <10% by 3 months compared with 9 and 11% of those treated in the two nilotinib arms. In all groups, failure to achieve EMR was associated with increased risk of disease progression and a lower overall survival, again further verifying the findings from earlier studies. It was also shown in this study that patients considered to be high risk, as denoted by high Sokal scores at diagnosis, were significantly more likely to achieve EMR with nilotinib than with imatinib. A high-risk Sokal score was associated with EMR failure in 56% of those on imatinib compared with 14 and 18% in of those on nilotinib, suggesting that certain high-risk patients would particularly benefit from first-line nilotinib treatment. Estimated PFS for patients reaching EMR were statistically different in both nilotinib arms (95.2 vs 82.9% for nilotinib 300 mg twice daily and 96.9 and 89.0% for nilotinib 400 mg twice daily) as well as in the imatinib arm (97.7 and 82.6%).

The MD Anderson Cancer Center group compared four different TKI modalities, including imatinib at 400 and 800 mg doses, dasatinib and nilotinib, and found that EMR was predictive for EFS and OS regardless of the treatment modality [22]. They also identified a significantly higher proportion of patients in the imatinib standard 400-mg dosing group who had a poor response at 3 months.

Finally, a recent smaller Scandinavian study comparing imatinib and dasatinib showed similar results, with superior early molecular and cytogenetic responses in the dasatinib group. Ninety-five percent of patients treated with dasatinib achieved EMR, compared with 71% of those treated with imatinib. The molecular and cytogenetic targets were eventually achieved in most patients in the imatinib group after approximately 18 months; however, MR4.5 remained consistently superior in the dasatinib group [23].

## 3- vs 6-month EMR Assessment

Neelakanthan et al. attempted to improve on the prognostic information provided by the traditional 3-month EMR assessment by incorporating reaching a *BCR-ABL1* transcript level of <1% at 6 months as an additional prognostic indicator [24•]. They looked at 274 patients treated either with first-line imatinib or dasatinib. Eleven percent of patients had a 3-month cutoff of <10% with a 6-month cutoff of >1%, with this group showing overall results similar to those in the group where both markers were below the defined cutoffs. Furthermore, although only accounting for 2% of patients, those with levels >10% at 3 months but <1% at 6 months had outcomes similar to those with results higher at both time points. As such, they concluded that an assessment at 3 months alone is sufficient for accurate prognosis and reaffirmed that adding data from molecular assessment at 6 months offers little further prognostic information. However, Kim et al. reported conflicting findings in their study of 320 patients treated with imatinib [25]. They showed that a group of patients who failed to achieve EMR but did achieve reduction in transcript level to <1% at 6 months had similar outcomes to those with EMR, although the later group only accounted for 6% of patients. Those who failed to achieve a response at 3 and 6 months had worse PFS compared to patients who did not achieve an EMR at 3 months but then at 6 months achieved a level between 1 and 10% as well as to those who achieved EMR at 3 months. This suggested that it was in fact possible to identify a “good-risk” group of patients of those who fail to achieve EMR, by looking at transcript levels at 6 months.

Hanfstein et al. reanalyzed data from the 2012 CML IV study to determine whether the 10% reduction in BCR-ABL transcript level was more significant at 3 or 6 months [26••]. They found that 8-year PFS and OS rates were comparable; however, the 3-month landmark was found to be significantly more sensitive and the 6-month landmark more specific, as shown by identification of a smaller number of high-risk patients and fewer cases of progression using the 6-month landmark, which was found to be less than half as sensitive as the 3-month landmark. In addition, a treatment intervention at 6 months might also prevent less progressions due to the delay of 3 months. However, transcript level halving time at 3 months was found to be as sensitive as the 3-month EMR but with improved specificity to predict progression.

## Importance of Halving Time

Recent focus has switched to whether the rate of decline in *BCR-ABL1* transcript level has more prognostic significance, partially in view of the fact that a significant proportion of patients who fail to meet standard EMR criteria goes on to achieve good outcomes. Branford et al. reported on 95 patients who failed to achieve EMR with imatinib treatment [27••]. Patients were divided into two groups dependent on having a halving time above or below 76 days. Seventy-four patients were identified as having a *BCR-ABL1* halving time of <76 days and were found to have significantly better outcomes than the 21 patients who did not meet this criterion, with an OS of 95 vs 58% (*P* = 0.0002) and a PFS of 92 vs 63% (*P* = 0.008). They emphasized the heterogeneous outcomes seen in patients with EMR failure and suggested that the use of halving times may better define the group of patients at the highest risk in whom alternative treatment strategies should be considered.

Similarly, the German group measured the rate of *BCR-ABL1* decline over the first 3 months of treatment using beta glucuronidase as the control gene in a group of 408 patients [28••]. They highlighted that the *BCR-ABL1* transcript level at 3 months is dependent on the initial tumor burden as well as the treatment-related rate of decline. They also suggested that the use of *ABL* as a reference gene for *BCR-ABL1* transcript monitoring is problematic as *BCR-ABL* is incorporated into the total *ABL* quantitation. They measured a 3-month reduction ratio, defined as the ratio of the transcript level at 3 months and that at diagnosis, and found that a half log reduction in *BCR-ABL* transcript levels was the most precise predictive cutoff. They found an OS of 98% in patients with a half log reduction, compared with 83% in those who did not achieve this rate of reduction. They suggested the use of an initial *BCR-ABL* transcript level but subsequent use of beta glucuronidase to allow for accurate individual determination of *BCR-ABL* decline.

Finally, a Japanese study recently looked at the halving time in patients treated with dasatinib [29]. They included 52 patients and calculated halving time using transcript levels before treatment and after 3 months and the number of treatment days between these two points. Ninety percent of patients included achieved EMR by standard criteria. They found that a shorter halving time of less than 14 days was associated with increased likelihood of achieving MMR (97 vs 50%). In addition, 89% of the patients in the shorter halving time group achieved MR4 by 18 months compared with only 29% in the longer halving time group, and this was the only factor that predicted achieval of deep molecular response by 18 months.

## EMR and TKI Discontinuation

A rapidly developing area in the treatment of patients with CML is the identification of patients in whom there is the potential to discontinue TKI treatment. Studies have estimated that between 40 and 60% of patients who stop imatinib are able to maintain MMR [30–32]. EMR, as a measure of the likelihood of achieving deep molecular response, can also predict a patient’s chance of attempting treatment discontinuation. Branford et al. collected data on 423 newly diagnosed patients treated with imatinib, with the primary aim of determining the proportion of patients who meet the criteria for discontinuation [33•]. They identified a cumulative incidence of stable MR4.5 of 36.5% after 8 years of imatinib treatment; however, only approximately a third of these patients maintained this for the required 2-year period. In multivariate analysis, they found that gender and *BCR-ABL1* reduction at 3 months were the only independent factors predictive of stable MR4.5. MMR at 3 months was associated with subsequent stable MR4.5 of 78.2% at 8 years; however, this was not surprisingly only achieved in 10% of patients.

## Impact of Early TKD Mutation at 3 months

Mutations in the *BCR-ABL1* kinase domain (KD) affect a significant proportion of CML patients and have been associated with primary or acquired (refractory disease following an initial response) resistances to TKIs [34–36]. Such resistance may emerge at any time during TKI therapy and identify those at high risk of disease progression [37].

While nilotinib and dasatinib are active against most imatinib-resistance mutations, other mutations may also confer resistance (thus a poor response) to second-generation TKIs. The detection of such mutations following imatinib resistance is therefore critical to ensure appropriate second-line or third-line drug selection.

In a cohort of 121 CP-CML patients presented at the 2015 American Society of Hematology, we reported that next generation sequencing (NGS) can reliably detect low-level KD mutations otherwise not detectable by Sanger sequencing (SS) [38]. In particular, we found that NGS may detect the appearance of KD mutations as early as 3 months after TKI initiation in patients who failed to respond. All patients transformed to advanced phase following mutation detection, suggesting that early screening for KD mutations by NGS at 3 months might be useful to identify those at higher risk of transformation in addition to using EMR.

## Conclusion

Although several groups have reported the importance of the EMR milestone, clinical intervention based on it remains controversial partly due to its low specificity to predict progression. This may be partially improved by using the rate of decline in *BCR-ABL1* transcript level (halving time or velocity of ratio reduction) although standardization would be needed. The results of ongoing randomized clinical trials looking at early switch of TKI based on EMR will also be useful to define clinical strategies. In a small subset of patients, early identification of KD mutations at 3 months may also prove useful in clinical practice.
